# CD73 mediated host purinergic metabolism in intestine contributes to the therapeutic efficacy of a novel mesenchymal-like endometrial regenerative cells against experimental colitis

**DOI:** 10.3389/fimmu.2023.1155090

**Published:** 2023-04-25

**Authors:** Bo Shao, Shao-hua Ren, Zhao-bo Wang, Hong-da Wang, Jing-yi Zhang, Hong Qin, Yang-lin Zhu, Cheng-lu Sun, Yi-ni Xu, Xiang Li, Hao Wang

**Affiliations:** ^1^ Department of General Surgery, Tianjin Medical University General Hospital, Tianjin, China; ^2^ Tianjin General Surgery Institute, Tianjin Medical University General Hospital, Tianjin, China; ^3^ School of Basic Medical Sciences, Tianjin Medical University, Tianjin, China

**Keywords:** inflammatory bowel diseases, purinergic metabolism, CD73, human endometrial regenerative cells, intestinal barrier, mucosal immune responses

## Abstract

**Background:**

The disruption of intestinal barrier functions and the dysregulation of mucosal immune responses, mediated by aberrant purinergic metabolism, are involved in the pathogenesis of inflammatory bowel diseases (IBD). A novel mesenchymal-like endometrial regenerative cells (ERCs) has demonstrated a significant therapeutic effect on colitis. As a phenotypic marker of ERCs, CD73 has been largely neglected for its immunosuppressive function in regulating purinergic metabolism. Here, we have investigated whether CD73 expression on ERCs is a potential molecular exerting its therapeutic effect against colitis.

**Methods:**

ERCs either unmodified or with CD73 knockout (CD73^-/-^ERCs), were intraperitoneally administered to dextran sulfate sodium (DSS)-induced colitis mice. Histopathological analysis, colon barrier function, the proportion of T cells, and maturation of dendritic cells (DCs) were investigated. The immunomodulatory effect of CD73-expressing ERCs was evaluated by co-culture with bone marrow-derived DCs under LPS stimulation. FACS determined DCs maturation. The function of DCs was detected by ELISA and CD4^+^ cell proliferation assays. Furthermore, the role of the STAT3 pathway in CD73-expressing ERCs-induced DC inhibition was also elucidated.

**Results:**

Compared with untreated and CD73^-/-^ERCs-treated groups, CD73-expressing ERCs effectively attenuated body weight loss, bloody stool, shortening of colon length, and pathological damage characterized by epithelial hyperplasia, goblet cell depletion, the focal loss of crypts and ulceration, and the infiltration of inflammatory cells. Knockout of CD73 impaired ERCs-mediated colon protection. Surprisingly, CD73-expressing ERCs significantly decreased the populations of Th1 and Th17 cells but increased the proportions of Tregs in mouse mesenteric lymph nodes. Furthermore, CD73-expressing ERCs markedly reduced the levels of pro-inflammatory cytokines (IL-6, IL-1β, TNF-α) and increased anti-inflammatory factors (IL-10) levels in the colon. CD73-expressing ERCs inhibited the antigen presentation and stimulatory function of DCs associated with the STAT-3 pathway, which exerted a potent therapeutic effect against colitis.

**Conclusions:**

The knockout of CD73 dramatically abrogates the therapeutic ability of ERCs for intestinal barrier dysfunctions and the dysregulation of mucosal immune responses. This study highlights the significance of CD73 mediates purinergic metabolism contributing to the therapeutic effects of human ERCs against colitis in mice.

## Introduction

Inflammatory bowel disease (IBD), including ulcerative colitis (UC) and Crohn’s disease (CD), is a chronic, idiopathic, relapsing, and inflammation-induced gastrointestinal (GI) tract disease characterized by intestinal mucosal ulceration (1). Patients with IBD suffer diarrhea, abdominal pain, recurrent haematochezia, and weight loss, severely impacting patients’ quality of life (2). Furthermore, chronic inflammation also increases the risk of colorectal carcinoma (3). The etiology of IBD remains enigmatic, but the hypothesis is generally believed that aberrant purinergic metabolism can cause barrier dysfunction of the epithelium, leading to loss of mucosal immune system tolerance to intestinal antigens (4–6). The mucosal immune system in the gut is a strictly controlled balance between innate and adaptive effector responses and negative regulation, alteration of the balance between immunity for protectiveness and tolerance to autoantigens and symbiotic bacteria have been emphasized as pathogenic factors in IBD (7).

The current conservative treatment of IBD includes antibiotics, amino-salicylate, corticosteroids, anti-tumor necrosis factor (TNF) inhibitors, and immunomodulators (8). However, long-term use can lead to severe complications such as hepatotoxicity, opportunistic infections, autoimmunity, and malignant tumors (9). About 15% of UC patients and 40% of CD patients in severe conditions require intestinal resection within 20 years of pathogenesis (10, 11). Therefore, developing the application of non-invasive therapy in medical interventions is imperative. Stem cell therapy has appeared in managing IBD with regeneration, low immunogenicity, and immunomodulatory characteristics (12). Having shown therapeutic efficacies against animal experiments of IBD, mesenchymal stem/stromal cells (MSCs) have been endorsed by the European Medicines Agency for the therapy of CD following the phase I/II clinical trial (13, 14). However, the invasive procedure for harvesting the cells, and the application of MSCs, including adipose-derived stem cells (ADSCs) and bone marrow-derived MSCs (BMSCs), is limited (15).

An emerging mesenchymal-like endometrial regenerative cells (ERCs) derived from women’s menstrual blood retained fundamental MSC properties in self-renewal, multi-lineage differentiation, and immune regulation. Compared to MSCs obtained from other adult tissues, such as bone marrow, amniotic fluid, and adipose tissue, ERCs could be obtained through a safe, simple, non-invasive, low-cost procedure with fewer ethical issues. Moreover, ERCs exhibited higher proliferation rates than MSCs derived from adult bone marrow, suggesting they could have more significant potential for clinical applications (16). ERCs mediated systemic immunosuppression and were used in some preclinical and clinical studies to treat various diseases, such as experimental graft-versus-host disease (GVHD), pulmonary fibrosis, liver fibrosis, myocardial fibrosis infarction, renal ischemia-reperfusion injury and COVID-19 (16, 17). Mechanism of interaction depended on direct cell-cell contact and/or secretion of immunosuppressive factors by ERCs such as nitric oxide (NO), indoleamine 2,3 deoxygenase (IDO), prostaglandin E2 (PGE2), tumor necrosis factor-α stimulated gene 6 (TSG-6) and stromal cell-derived factor-1 (SDF-1) (18, 19). For example, Jiang et al. have demonstrated that ERCs significantly reduced apoptosis and promoted cell regeneration in myocardial infarction (MI) rats (20). This was regulated by secreted cytokines, including EGF, PDGF, nitric oxide (NO) and TGF-β, to activate the AKT/extracellular signal-regulated kinase 1 and 2 (ERK 1/2)/STAT-3 signaling pathway. Furthermore, PGE2 is an important player in chronic inflammation, regulating DC, NK and lymphocyte functions. Aleahmad M, et al. showed that PGE2 secreted by ERCs was involved in the inhibition of T cell proliferation by diminishing the synthesis of IL-2 and IL-2 receptors in the co-culture system (21). The safety and efficacy of ERCs have been tested, but the development of ERCs as a therapy for IBD is still in its infancy. Its potential mechanisms remain to be explored *in vitro* and animal experiments.

Ecto-5’-nucleotidase (CD73), as an immunophenotypic marker of ERCs attached to the plasma membrane, was the main enzyme implicated in the production of extracellular adenosine (ADO) in purinergic metabolism (22). During inflammation, adenosine triphosphate (ATP) was released from the cytoplasm into the extracellular matrix, which activates and recruits immune cells, augmenting the inflammatory response. Extracellular ATP could rapidly hydrolyze into adenosine monophosphate (AMP) by CD39, but the transformation of AMP to ADO is regulated by CD73 (23). ADO has a significant immunosuppressive effect and functions primarily to counteract pro-inflammatory ATP in tissue healing and inflammation. Additionally, the ADO could bind to four ADO receptors: A1, A2a, A2b, and A3 receptors (24). As a result, CD73 can perform biological functions by itself and *via* the interaction between the generated ADO and ADO receptors that work together (25). For example, CD73 deficiency developed markedly more severe colitis, exhibited unresolved inflammation, and produced high levels of the pro-inflammatory cytokines TNF-α and IL-1β compared to wild-type mice (26). Furthermore, CD73-ADO signaling has been shown to alleviate immune-mediated tissue damage and prevent excessive immune reactions in colitis through binding to the A2a and A2b receptors (27, 28).

ERCs are thought to be a defender against excessive pro-inflammatory responses. Several immunosuppressive mechanisms have been identified for ERCs, which can be used to modulate inflammation in a specified environment. However, no pertinent studies have reported focusing on evaluating CD73’s contribution to mediating the immunomodulatory effects of ERCs in the attenuation of colitis. Given the promising immunomodulatory capability of CD73, exploring its effects in ERCs was proposed. In this study, ERCs with high levels of CD73 on their membrane surface could convert AMP to immunosuppressive ADO. We investigated the contribution of CD73 to ERC-mediated alleviation of DSS-induced colitis in mice. We explored the molecular basis of CD73-expressing ERC’s contribution to the intestinal epithelial barrier and associated immune cells.

## Materials and methods

### ERCs isolation, culture, and identification

Human menstrual blood was collected from healthy female volunteers, 20 to 30 years old, after regular deliveries with written consent. The procedure for menstrual blood sample collection was approved by the Medical Ethics Committee of Tianjin Medical University General Hospital (IRB2021-KY-347, Tianjin, China). Human ERCs were isolated, as described previously (29, 30). On the first day of the menstrual cycle, menstrual blood was collected from healthy female volunteers (20-30 years old) using a sterile menstrual cup. The samples were mixed with equal volume of phosphate-buffered saline (PBS) containing 100 U/ml penicillin, 100 mg/ml streptomycin and 2 mM, 0.25 mg/ml amphotericin B, and ethylenediaminetetraacetic acid (EDTA). The menstrual blood was filtered with 200 μm mesh filter and suspended in Dulbecco ‘s Modifed Eagle Medium/Nutrient MixtureF-12 (DMEM-F12) (Hyclone, USA), and then the harvested suspension was added to Ficoll solution gradient, and centrifuged at 2000rpm for 20 minutes to obtain monocyte layer cells. The harvested cells were resuspended in DMEM-F12 complete medium supplemented with 15% fetal bovine serum (Corning, New Zealand) added 1% penicillin/streptomycin (Solarbio, Beijing, China), and then inoculated in 6-well plates at 5% CO2 and 37°C. The culture medium was changed twice a week, and the ERCs were passaged when subconfluent with 0.1% trypsin solution (Solarbio, China). ERCs were obtained with fluorescent antibodies for phenotype identification, including surface marker CD79a-PE, HLA-DR-FITC, CD105-PE-Cyanine7, and CD90-PE from eBioscience.

### Preparation of ERCs with CD73 ablation and measurement of the adenosinergic enzymatic activity of CD73 on ERCs *in vitro*

To knock out CD73 (NT5E, NM_002526) in ERCs, lentiviral transfection of ERCs was conducted according to the manual provided by GeneChem Inc., Shanghai, China (The vector conducted as U6-sgRNA-EF1a-Cas9-FLAG-P2A-Puromycin.). Lentiviral transfection was done in a biological safety cabinet with an optimum multiplicity of infection (MOI=35). Then, ERCs were screened twice with 2μg/mL puromycin (Solarbio, China) after transfection. ERCs from Passages 5-7 were used for subsequent experiments. The generated CD73^-/-^ERCs were tested by flow cytometry and western blot before treatment to ensure cell purity and quality. After three washes in glycine buffer, the treated cells were used to test the enzymatic activity with the Phosphate Assay Kit (ab270004, Abcam). In brief, 100 mM ADO monophosphate (A8300, Solarbio) was added to the wells, followed by 1h incubating at 37°C. Then, the supernatant was collected separately and added to a 96-well plate, and a 0.25 volumes mixture (PiColorLock reagent and Accelerator) was added. After 5 min of complete reaction, a 0.1 volumes stabilizer was added. Finally, 30 min later, the plate is counted at 590 nm through a microplate reader (Safire2, TECAN).

### DSS-induced colitis in mice

All the animal experiments were conducted according to the guideline of the Chinese Council on Animal Care and approved by the Animal Ethical and Welfare Committee of Tianjin Medical University General Hospital (IRB2021-DWFL-405, Tianjin, China). Specific pathogen free male BALB/c and C57BL/6 mice aged 7 weeks (weighing 20–22 g) were purchased from China Food and Drug Inspection Institute (Beijing, China). A week of adaption to the new environment was followed by random assignment to experimental groups. In the present study, BALB/c mice were randomly assigned to 4 groups: Control group, Untreated group, ERCs-treated group, and CD73^-/-^ERCs-treated group (n=6 in each group). All experimental groups for colitis were induced with 3% DSS (36-50 kDa; 60316ES60, YEASEN) supplemented in the drinking water for 7 days and then received normal water for the following 3 days. For treatment, ERCs or CD73^-/-^ ERCs (5^th^-7^th^ generation) were suspended in phosphate buffered saline (PBS) and injected intraperitoneally experimental mice (1×10^6^ cells per mice) on days 2, 5, and 8 according to the protocols (31, 32). All other treatments were also administered with the volume of PBS (200μL) as the vehicle group simultaneously. Body weight changes were measured daily during the experiment. On day 10, the mice were euthanized, and the entire colon was harvested. The length of the colon was measured and gently rinsed with cold PBS. Two 0.5 cm long sections of the distal colon were then taken for histological evaluation and immunohistochemical staining. The residual colon tissue samples were cut longitudinally, flushed with cold PBS for intestinal contents and snap-frozen for subsequent molecular biology experiments. Mesenteric lymph nodes (MLNs), spleen, and serum samples were collected from sacrificed mice for further experiments.

### ERCs labeling and bioluminescence imaging

To track transplanted cells *in vivo*, ERCs of passage 2 were transfected with a lentiviral vector (Ubi-MCS-firefly_Luciferase-IRES-puromycin, GeneChem, China) that carried the ubiquitin promoter encoding luciferase with an optimum multiplicity of infection (MOI = 50). Then, ERCs were screened twice with 2μg/mL puromycin after transfection. As previously described (13), the Fluc activity of different ERCs numbers and the fate of transplanted ERCs were observed in living colitis mice using the IVIS Lumina Imaging System (Xenogen Corporation, Hopkinto, MA). After intravenous/intraperitoneal injection of the reporter probe D-fluorescein (150 mg/kg, IL0230, Solarbio), the animals were photographed with the IVIS Lumina imaging system for 1-10 min. Bioluminescence signal intensity images were analyzed and processed using live image software (Xenogen, Inc.).

### Histopathological analysis

Briefly, two 0.5 cm long sections of the distal colon in each mouse were first fixed in 10% neutral formalin-fixed for 48h, dehydration, paraffin embedding, and then sliced into a thickness of 3μm with an ultra-microtome (LEICA, Germany) for hematoxylin-eosin (H&E) staining. The severity of colonic epithelial damage and inflammatory cell infiltration was scored blinded to minimize observer bias, as mentioned previously (33). Briefly, the colonic epithelial damage scores were as follows: 0, normal; 1, hyperplasia, goblet cell loss, and irregular crypt foci; 2, mild to moderate crypt foci loss (10–50%); 3, severe crypt foci loss (50–90%); 4, complete crypt foci loss, intact epithelium; 5, small to medium ulcers (<10 crypt widths); 6, large ulcers (≥10 crypt widths). Inflammatory infiltration was scored separately for the mucosa (0, normal;1, mild; 2, moderate; 3, severe), submucosa (0, normal; 1, mild to moderate; 2, severe), and muscle/serous layer (0, normal; 1, moderate to severe). Total scores ranged from 0 to 12 based on epithelial damage and inflammatory infiltration.

### Immunohistochemical and goblet cell staining

To examine the dendritic cell (DCs) infiltration in colons of different groups, immunohistochemistry was performed as previously mentioned (34). Briefly, paraffin-embedded tissues are thermally fixed, dewaxed, rehydrated, antigen retrieval, blocked, and subsequently processed according to the manufacturer’s instructions. Firstly, colonic tissue slices were heat-fixed for 90 min at 60°C, deparaffinized in xylene for 40 min, then rehydrated in 100%, 90%, 80%, and 70% ethanol, respectively, for 5 min and washed three times with PBS at room temperature for five minutes each time. Antigen retrieval was performed with tris-EDTA antigen retrieval solution (C1038, Solarbio) under 100°C for 15 min in the microwave oven. The slices were incubated with 3% H_2_O_2_ for 25 min in the dark, blocked by 5% goat serum for 30 min, and stained with anti-CD11c (1:300, A1508, ABclonal) overnight at 4°C, washed with PBS-0.05% Tween20 (PBST). Then, HRP-labeled goat anti-rabbit IgG polymer (ZLI-9018, ZSGB-BIO) was added to slices for 30 minutes and washed three times with PBST at room temperature for five minutes each time. In the color reaction, 3,3’-Diaminobenzidine (TMB) was added for 40 s, followed by hematoxylin counterstaining for 30 s. To dehydrate the slices, they were immersed in 50%, 70%, 80%, 90%, and 100% ethanol and xylene for 5 minutes each. Finally, the CD11c^+^ cell rate was identified and calculated using Image J software (35).

For goblet cell staining, distal colon tissues were fixed in Carnoy’s fixative and cut into 3μm paraffin slices. After dewaxing and rehydration, the slices were stained with an Alcian Blue Stain Kit (G1560, Solarbio). Briefly, the slices were stained with Alcian Blue solution and then washed with distilled water (dH_2_O) for 2 min. Then, the nuclei were stained with nuclear solid red for 5 min and rinsed in dH_2_O for 1 min. The slices were then dehydrated, transparent, and sealed with a neutral resin. Alcian Blue^+^ mucin granules in the crypts were identified by counting in well-defined crypts, and goblet cells were normalized by the total number of IECs (stained nuclei) lining the crypts (36). In most cases, goblet cell numbers were counted from 20 random colonic glands from each mouse with 6 mice in each group (*i.e.* 120 crypts/group).

### Transmission electron microscopy imaging

To examine the contribution of CD73^-/-^ERCs on the ultrastructure of epithelium, the colons were collected and fixed immediately in 4% glutaraldehyde (P1127, Solarbio) for 24h at 4°C, rinsed in 0.1 M PBS, fixed in 1% osmium tetroxide for 3h at 4°C, rinsed in 0.1M PBS, dehydrated in an ascending alcohol series, soaked in propylene oxide, embedded in Epon 812 (45345, Sigma), sliced on an ultramicrotome (LKB-V, Pharmacia, Sweden), stained with uranyl acetate and lead citrate, and imaged with a transmission electron microscope (H7800; HitachiLtd., Japan).

### Intestinal permeability

The tracer fluorescein isothiocyanate-dextran (FITC-dextran) was utilized to evaluate intestinal permeability *in vivo*, as previously mentioned (37). Briefly, mice were deprived of food 6h prior to administering 4kDa FITC-dextran (0.6 mg/g body weight, FD4, Sigma) intragastrically in 100μL PBS and deprived of both food and water 4h after oral administration on the day of sacrifice. Hemolysis-free serum was collected retro-orbitally from mice, and fluorescence intensity was measured with an F-4500 Hitachi fluorescence spectrophotometer (excitation, 488 nm; emission, 520 nm). Prepare a standard curve for FITC-dextran by gradient diluting a known amount of FITC-dextran in mice serum.

### MLN cell preparation and flow cytometry

To analyze the quality and quantity of the immune response during intestinal inflammation, single cell suspensions were obtained from the MLNs, as previously mentioned (38). Briefly, single cell suspensions were obtained from MLNs by disrupting them mechanically through a 40μm cell strainer (Sigma). To block the nonspecific binding of the antibodies, the cell suspension was incubated with anti-CD16/32 mAb (eBioscience) for 10 min. It was stained with Zombie NIR™ (BioLegend, USA) to identify live/dead cells on ice for 15 min. For surface staining, cells were incubated with fluorescent antibodies, including FITC-CD4, PE-CD25, APC-CD11c, FITC-MHC-II, and PE-CD86 for 50 min in the dark at 4°C. For intracellular staining, cells were washed with cold PBS after surface staining, then fixed and permeabilized using a fixation/permeabilization kit (ThermoFisher Scientific). Cells were incubated with fluorescent antibodies of PE-IFN-γ, Percp-cyanine5.5-IL-17A, and APC-Foxp3 for 50 min in the dark at 4°C. As for the intracellular staining of IFN-γ and IL-17A, the cell stimulation cocktail plus protein transport inhibitor (Thermo Fisher Scientific) was added and incubated in an incubator at 37°C with 5% CO2 for 6h, staining as described above. All samples were rinsed three times with cold PBS and then analyzed with a BD FACSCanto™II flow cytometer and FlowJo. All fluorescent antibodies used in the present study were from eBioscience.

### Bone marrow-derived DC isolation and co-culture

BMDCs were obtained from BALB/c mice bilateral femur and tibia, as previously described (39). To assess the contribution of CD73^-/-^ERCs on DC activation, ERCs and immature DCs (1×10^6^/mL) were seeded into the upper and lower layers, respectively at a 1:10 ratio, of the 12 well Transwell^®^ plates (0.4μm pore size, CORNING) at day 10. Then, the Transwell^®^ system was stimulated for 24h with 200ng/mL LPS (Sigma-Aldrich). In the present co-culture system, immature DCs were randomly assigned to 4 groups: Control, LPS, LPS+ERCs, and LPS+CD73^-/-^ERCs (n=6 in each group). Phenotype analysis, protein expression, and cytokine secretion were assessed on DCs isolated from adherent ERCs. Isolated DCs were CD11c^+^ (>80%).

### CD4^+^T cell proliferation assay

LPS-stimulated DC were co-cultured with ERCs or CD73^-/-^ERCs in the Transwell^®^ system for 24h, washed with PBS, and then co-cultured with CD4^+^ lymphocytes purified by CD4 (L3T4) MicroBeads (130-117-043, Miltenyi biotec) at 1:10 ratio in a total volume of 0.2ml medium in 96-well U-bottom plates. The proliferative response was evaluated by Ki-67 staining through flow cytometry after 72 hours identified by the percentage of Ki-67^+^ cells.

### Enzyme-linked immunosorbent assay

All samples were readjusted to the same protein concentration prior to assay, and the colon homogenate and cell supernatants of TNF-α, IL-6, IL-1 β, and IL-10 in each group were assessed by the ELISA kit (DAKEWE, China). The optical density (OD) value was measured with a microplate reader.

### Western blot

Snap-frozen colon tissues and harvested cells were lysed in RIPA lysis buffer (R0010, Solarbio) containing protease and phosphatase inhibitor cocktail (P1261, Solarbio). The concentrations of proteins were determined using the BCA Protein Assay Kit (PC0020, Solarbio). The protein samples (20μg/40μg per lane) were separated by 7.5% or 15% SDS polyacrylamide gel and transferred to polyvinylidene fluoride membranes (0.45 μm; Millipore) using a semi-dry transfer method. The membranes were blocked with 5% skim milk in Tris-buffered saline with 0.1% Tween-20 (TBST) for 2h at room temperature, followed by incubation with primary antibodies against CD73 (1:1000, # 87661SF, CST), ZO-1 (1:1000, A0659, ABclonal), Occludin (1:1000, A12621, ABclonal), Claudin-1 (1:1000, A21971, ABclonal), p-STAT3 (1:2000, #9145S, CST), STAT3(1:1000, #12640S, CST) and β-Actin (1:1000, #4970S, CST) at 4°C overnight. The membranes were washed three times in TBST for 5 min each and incubated with HRP-conjugated anti-rabbit secondary antibody (1:5000, #7074S; CST) for 45 min. The target proteins were visualized using an enhanced chemiluminescence substrate kit (WP20005, Thermo Fisher Scientific). The densitometry of protein bands was quantified and analyzed using ImageJ software.

### Statistical analysis

All statistical analyses were performed using SPSS Statistics 26, for Windows (SPSS Inc., Chicago, IL, USA). The results were expressed as means ± standard error of mean (SEM). The normality of continuous variables was assessed using the Shapiro-Wilk test. Unpaired two-tailed Student’s t-tests between two groups and one-way ANOVA followed by LSD multiple comparison *post hoc* test was used to test differences among multiple groups. Statistical significance was set at *P < 0.05, **P < 0.01, ***P < 0.001 and ****P < 0.0001. GraphPad Prism v.8.0 (GraphPad Software) was used for statistical analysis.

## Results

### Phenotypic characterization of ERCs and catalytic activity of CD73 *in vitro*

**Figure 1:**
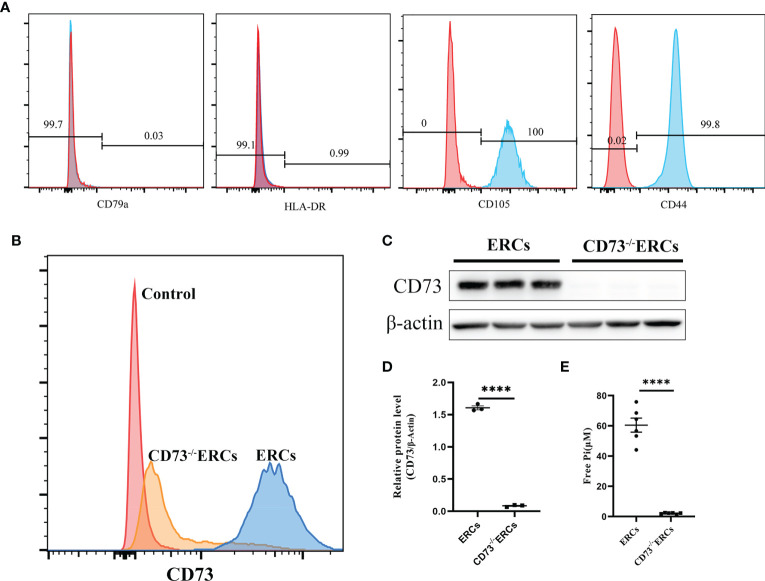
The characteristics of ERCs and the adenosinergic enzymatic activity of CD73 on ERCs *in vitro*. **(A)** ERCs were detected by flow cytometry to detect negative markers (CD79a and HLA-DR) and positive markers (CD44 and CD105) on cell surfaces for identification. **(B)** Flow cytometry reflected CD73 expression on the membrane of ERCs and CD73^-/-^ERCs. **(C)** Western blot analysis showed the differences in CD73 protein expression in ERCs and CD73^-/-^ERCs. **(D)** Gray value analysis with immunoblot based; CD73 intensity analysis was homogenized after comparing to β-actin (n=3). **(E)** Levels of inorganic phosphate (Pi) were measured after adding CD73 substrate (5′ AMP; 1 mM) (ERCs *vs*. CD73^-/-^ERCs: 60.46 ± 4.647 μM *vs*. 2.097 ± 0.1614 μM, P < 0.0001, n=6). Statistical analysis was done by using unpaired two-tailed Student’s t-tests. Data are presented as mean ± s.e.m (SEM). ****P < 0.0001, analyzed by unpaired t test. ERCs, endometrial regenerative cell; CD73^-/-^ERCs, ERCs transfected with lentivirus.

As illustrated in **Figure 1A**, flow cytometry analysis revealed that MSC-like ERCs were positive for phenotypic markers of CD105 (100%) and CD90 (99.8%) but were negative for CD79a (0.03%) and HLA-DR (0.99%). The CD73 expression can be detected in less than 4.2% of CD73^-/-^ ERCs post-transfection at passage 5 (**Figure 1B**). In addition, to further quantify the protein expression of CD73 after knockout, western blot analysis in **Figures 1C, D** showed an undetectable level of CD73 protein in CD73^-/-^ERCs as compared to that of unmodified ERCs (P <0.001).

To assess the enzymatic activity of CD73, which could hydrolyze AMP into ADO on the membrane surface of ERCs *in vitro*. The enzymatic activity of CD73 was quantized by calculating the free Pi as a paraproduct of AMP dephosphorylation. As shown in **Figure 1E**, the level of free Pi significantly declined in the CD73^-/-^ERCs group compared with that of the ERCs group (60.46 ± 4.647 μM in ERCs group *vs*. 2.097 ± 0.1614 μM in CD73^-/-^ERCs group, P < 0.0001). We also found that lentiviral vectors transfected with negative controls had no impact on the CD73 in ERCs (*data not shown*). The data suggested that ERCs, as the mesenchymal-like stromal cells, mainly express CD73 which is essential for ERCs in converting AMP to ADO. In addition, the positive rate of CD73 after screening by puromycin was less than 5% in ERCs post-transfection, which meets the needs of subsequent experiments.

### Knockout of CD73 reduced the therapeutic effects of ERCs in alleviating colitis

**Figure 2:**
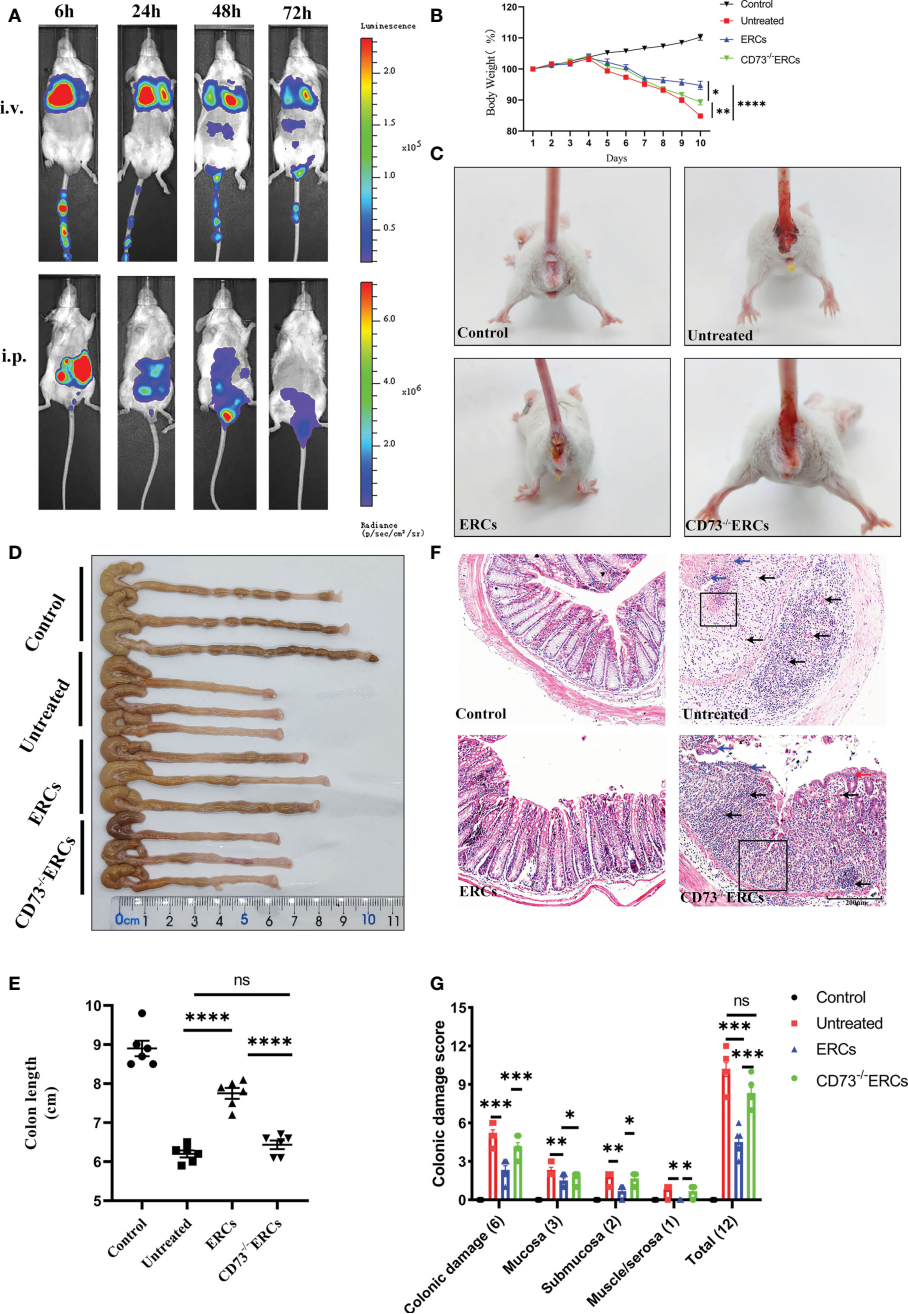
Knockout of CD73 reverses the therapeutic effect of ERCs on colitis. **(A)** *In vivo*, BLI of ERCs were tracked at 6, 24, 48, and 72 hours after intraperitoneal or tail intravenous injection in DSS-induced colitis mice. **(B)** Daily body weight changes in each group for 10 days (n = 6). **(C)** Representative pictures showed blood stools in BALB/c mice on day 10 after DSS induction. **(D, E)** Representative photographs of the colon in each group and the colon’s length in each group were analyzed on day 10 (n = 6). **(F)** Representative H&E-stained micrographs of the colon; blue arrows indicate epithelial detachment, red arrows indicate epithelial hyperplasia, black arrows indicate infiltrating inflammatory cells, and black boxes indicate the loss of enteric crypts (scale bar: 200 μm) (n=6). **(G)** Histopathology scores were calculated to assess colonic damage quantitatively. Data are presented as mean ± s.e.m (SEM). ns, no significance; *P < 0.05; **P < 0.01; ***P < 0.001; ****P <0.0001, analyzed by one-way ANOVA with LSD multiple comparison *post hoc* test.

Prior to exploring the effects of CD73 anchored on ERCs membranes, *in vivo* biodistribution of ERCs was investigated by IVIS imaging analysis *in vivo*. The cell lines with imaging reporter gene expression (Firefly Luciferase) were developed. Transduction of ERCs with imaging reporter gene did not affect the cell morphology, and BLI revealed a linear correlation between the ERCs number and luciferase signal (*data not shown*). The fluorescence signal of Luciferase-labeled ERCs was tracked at 6, 24, 48, and 72 hours after intraperitoneal or tail intravenous injection (**Figure 2A**). The intense signals were mainly presented in the lung and liver, and trace fluorescence signals were only shown around the intestine at 48 and 72 hours after ERC injection. Based on this result, ERCs were directly injected into the abdominal cavity in the animal study.

The critical role of CD73 in ERCs-mediated attenuation of acute colitis, which DSS induced was evaluated in BALB/c mice. Compared with untreated group, ERCs treatment significantly protected animals against DSS-induced body weight loss and bloody stool, while the effects of ERCs were weakened after knocking out CD73 (**Figures 2B, C**). The lengths of colons in the ERCs-treated group were significantly longer than those of untreated group due to severe intestinal inflammation (**Figures 2D, E**, p<0.0001). In contrast, deleting CD73 reduced the effect of ERCs in maintaining the length of the colon (CD73^-/-^ERCs-treated group *vs*. ERCs-treated group, P < 0.0001). Moreover, there was no significant difference in the colon length between PBS-treated and CD73^-/-^ERCs-treated colitis mice (**Figure 2E**). Pathological damage, including epithelial hyperplasia, goblet cell depletion, the focal loss of crypts, ulceration, and the infiltration of inflammatory cells, were significantly attenuated with the almost normal structure of the colon in ERCs-treated mice (**Figures 2F, G**). However, ERCs with CD73 knockout showed a weakened effect on improving pathological changes in colitis mice. Taken together, CD73 contributes to the therapeutic efficacy of ERCs against experimental colitis.

### Knockout of CD73 decreased ERC-induced restoration of the disrupted intestinal mucosal barrier

**Figure 3:**
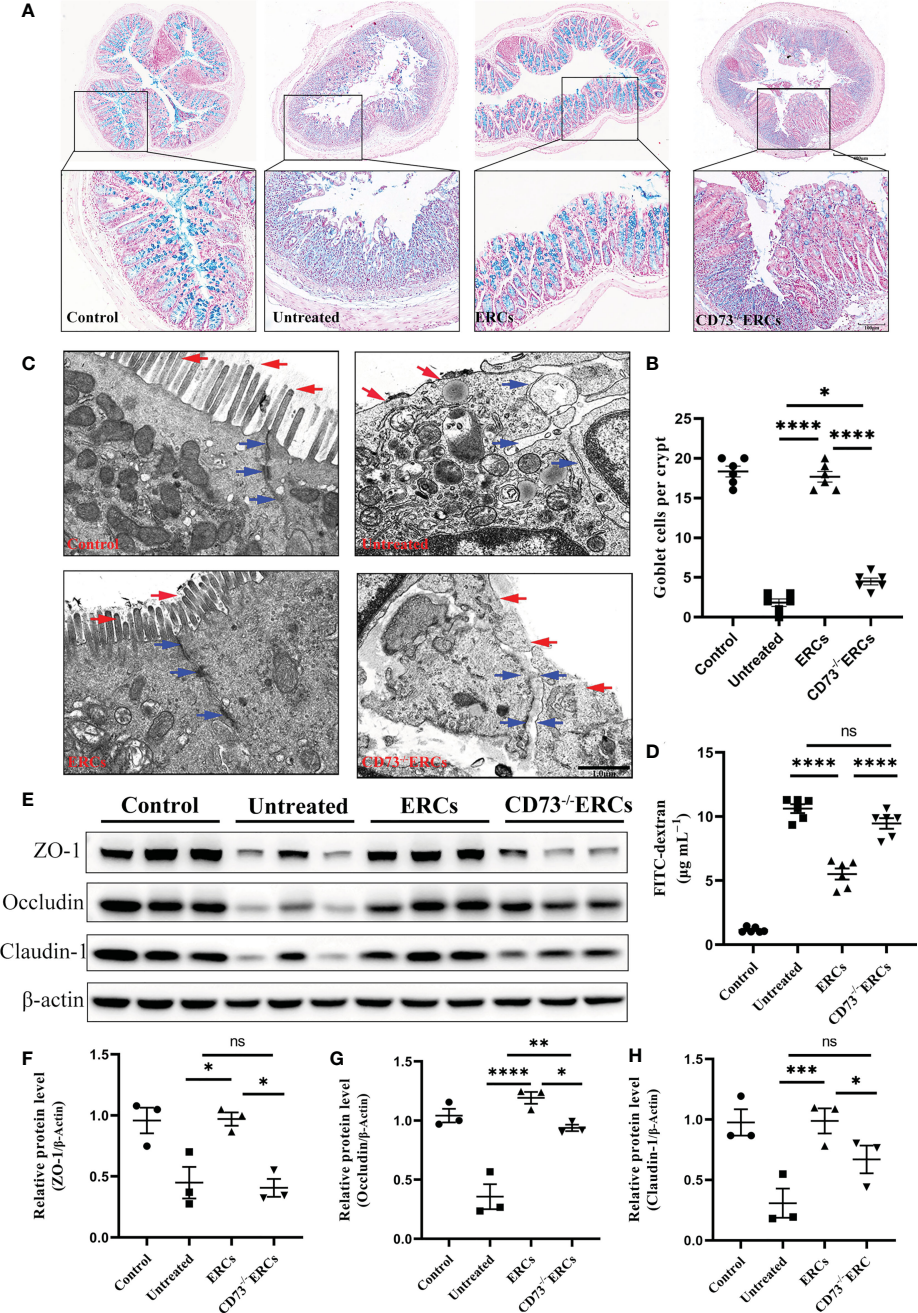
Knockout of CD73 reverses the effect of ERCs on intestinal barrier restoration in colitis. **(A)** Alcian blue staining showing goblet cells (blue) in colon tissue (Scale bars: 400 or 100μm). **(B)** Quantitative analysis indicates the number of goblet cells in colon tissue (n = 6). **(C)** Representative TEM images show colonic epithelial cells’ ultrastructure (scale bar:1μm). Red arrows indicate colonic cell microvilli, and blue arrows outline tight junctions and the intercellular space between two adjacent cells. **(D)** The concentration of serum FITC-dextran suggests the intestinal mucosa’s permeability in each group (n=6). **(E-H)** Quantitative analysis of intestinal interepithelial tight junction proteins ZO-1, Occludin, and Claudin-1 with β-actin as a reference in colon tissues measured by western blot (n=3). Data are presented as mean ± s.e.m (SEM). ns, no significance; *P < 0.05; **P < 0.01; ***P < 0.001; ****P <0.0001, analyzed by one-way ANOVA with LSD multiple comparison *post hoc* test.

Since CD73 could contribute to the protective function of the epithelial barrier, we evaluated the efficacy of CD73 expressed on ERCs in protecting colonic epithelial cells and intestinal barrier in DSS-induced inflammation. Alcian Blue staining was performed to quantify the number of goblet cells in the colon, which appeared blue under the microscope. As shown in **Figures 3A, B**, the number of colonic goblet cells in the ERCs-treated group was significantly higher than in both the untreated group (P < 0.0001) and the CD73^-/-^ERCs-treated group (P < 0.0001). The morphological ultrastructures of tight junctions (TJs; blue arrowheads) and microvilli (red arrows) were visualized under TEM (**Figure 3C**). Consistent with previous results, TEM demonstrated loss of TJ structures, irregular gaps and a considerable distance between adjacent colonocyte cells, and loose microvilli, representing the characteristics of intestinal epithelial barrier destruction in DSS-induced colitis mice. **Figure 3C** showed that the damaged TJ structure and microvilli were normalized in the ERCs-treated group. Conversely, CD73 knockout diminished the protective role of ERCs in this model (**Figure 3C**).

To further corroborate these findings, we designed an intestinal permeability assay by measuring serum FITC-dextran levels. Compared with other treatments, CD73 expressed by ERCs protected against systemic exposure to FITC-dextran after intraperitoneal administration in DSS-induced colitis mice (**Figure 3D**, P < 0.0001), suggesting the restoration of intestinal permeability. In contrast, the lack of CD73 affected the serum levels of FITC-dextran, confirming the critical role of CD73 in improving intestinal barrier function (**Figure 3D**, CD73^-/-^ERCs-treated group *vs*. ERCs-treated group, P<0.0001). Disruption of TJ resulted in increased permeability of the intestinal epithelium, and we investigated the expression associated proteins. Furthermore, intraperitoneal administration of ERCs to DSS-induced colitis mice significantly upregulated the levels of ZO-1 (**Figure 3F**, ERCs-treated group *vs*. untreated group, P<0.05), occludin (**Figure 3G**, ERCs-treated group *vs*. untreated group, P<0.0001), and claudin-1 (**Figure 3H**, ERCs-treated group *vs*. untreated group, P<0.001), which are TJ associated proteins that play a vital role in intestinal homeostasis. However, the impact of CD73^-/-^ERCs treatment was marginal (**Figures 3E-H** CD73^-/-^ERCs-treated group *vs*. ERCs-treated group, ZO-1, occluding and claudin-1, P<0.05). These results demonstrated the protective efficacy of ERCs expressing CD73 on the intestinal barrier in the context of DSS-induced colitis.

### Deletion of CD73 downregulated capacity of ERCs on CD4^+^ T cell differentiation *in vivo*

**Figure 4:**
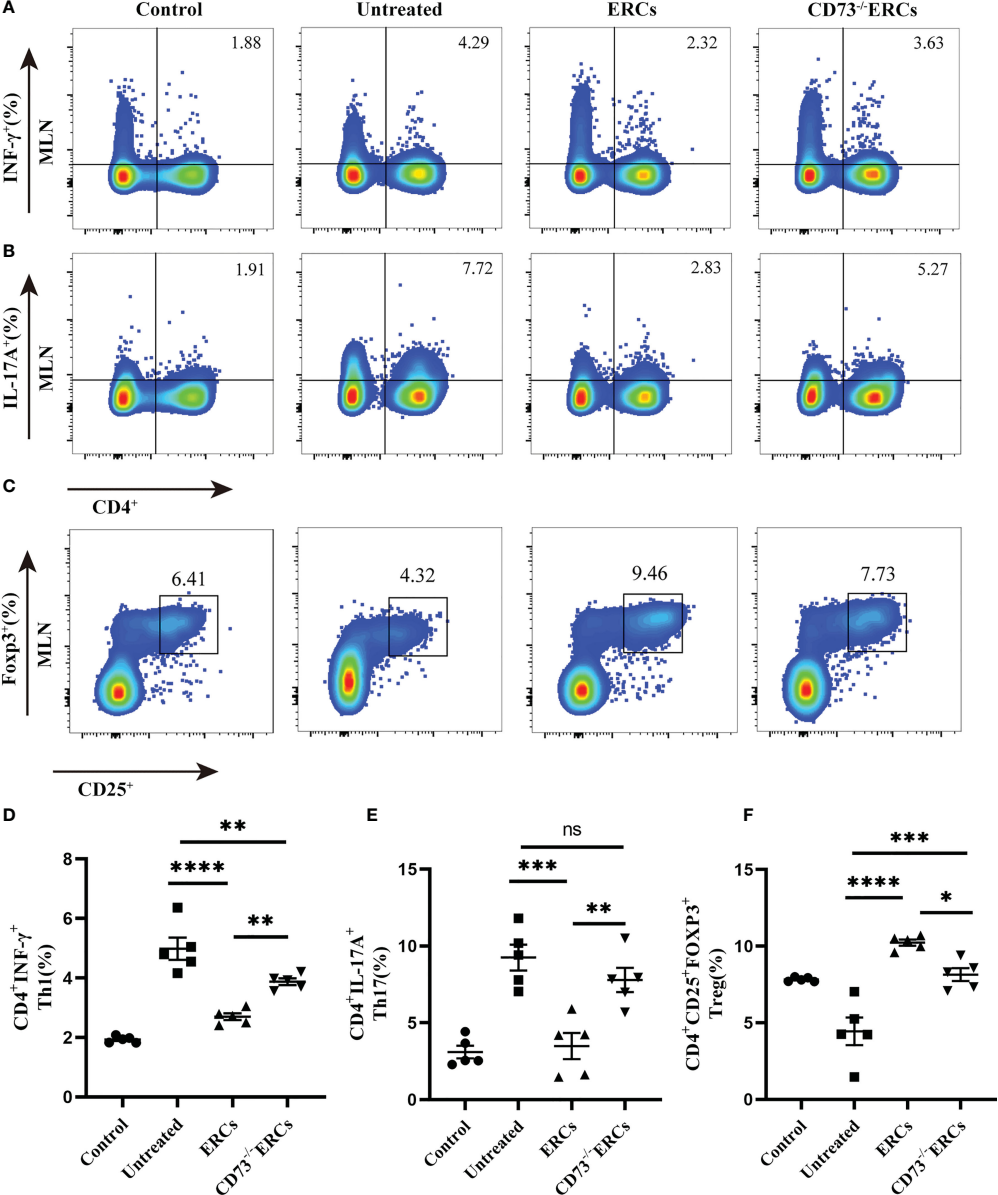
Knockout of CD73 reverses the effect of ERCs on CD4^+^T cell differentiation in colitis. MLN cells were collected on day 10 after DSS induction. To identify Th1 and Th17 cells, MLN cells were first incubated with a stimulation cocktail for 5 h, followed by staining with a fluorescent antibody. Flow cytometry plots and graph analysis of CD4^+^IFN-γ^+^ Th1 **(A)**, CD4^+^IL-17^+^Th17 **(B)**, and CD4^+^CD25^+^Foxp3^+^Tregs **(C)** in the MLNs from the Control, untreated, ERCs-treated and CD73^-/-^ERCs-treated groups. **(D-F)** Percentage of CD4^+^IFN-γ^+^Th1, CD4^+^IL-17^+^Th17 cells and CD4^+^CD25^+^Foxp3^+^Tregs (n=5). Data are presented as mean ± s.e.m (SEM). ns, no significance; *P < 0.05; **P < 0.01; ***P < 0.001; ****P <0.0001, analyzed by one-way ANOVA with LSD multiple comparison *post hoc* test.

As the key drivers of intestinal inflammation, T cell-mediated immune imbalance plays an essential role in the pathogenesis and development of IBD. We next assessed the relative proportions of Th cell and Treg subsets, including Th1 (CD4^+^IFN-γ^+^), Th17 (CD4^+^IL-17^+^) and Treg (CD4^+^CD25^+^Foxp3^+^) cells, in the MLNs of treated animals by flow cytometry (**Figure 4**). The percentage of CD4^+^IFN-γ^+^ and CD4^+^IL-17^+^ cells were decreased after ERCs treatment (Th1, P < 0.0001; Th17, P < 0.001), but this decrease was reversed by CD73^-/-^ERCs treatment (Th1, P < 0.01; Th17, P < 0.01), further suggesting that CD73 was implicated in regulating Th1 and Th17 cells in MLNs (**Figures 4A, B, D, E**). However, the proportions of splenic Th1 and Th17 cells were not significantly different between the ERCs-treated group and CD73^-/-^ERCs-treated group (*data not shown*). Similarly, as shown in **Figure 4**, the percentages of Treg were increased in the ERCs-treated group compared to those of untreated group (**Figures 4C, F**, Treg, P < 0.0001). CD73 knockout eliminated the effect of ERCs on increasing Treg populations (CD73^-/-^ERCs-treated group *vs*. ERCs-treated group: Treg, P < 0.05). These data suggested that CD73-expressing ERCs could significantly promote Treg differentiation in mice.

**Figure 5:**
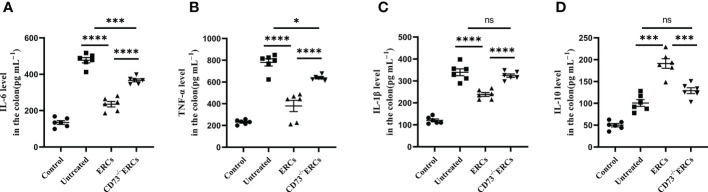
Knockout of CD73 reverses the effect of ERCs on cytokine profile in colitis. To evaluate the role of CD73 expression on ERCs in regulating immune response during intestinal inflammation at both quality and quantity levels, the production of pro-inflammatory cytokines (IL-1β, IFN-γ and TNF-α) and anti-inflammatory cytokines (IL-10) in colonic tissues was detected *via* ELISA assay. On day 10, the levels of pro-inflammatory **(A-C)** and anti-inflammatory **(D)** cytokines in the colonic tissues were analyzed (n=6). Data are presented as mean ± s.e.m (SEM). ns, no significance; *P < 0.05; ***P < 0.001; ****P <0.0001, analyzed by one-way ANOVA with LSD multiple comparison *post hoc* test.

The cytokines released by specific pro-inflammatory or anti-inflammatory T cells are the central mediators of inflamed mucosal lesions in IBD patients (40). To further support these findings, we measured cytokines profile in intestinal tissues by ELISA assay (**Figures 5A-D**). Apparently, CD73-expressing ERCs decreased the concentrations of IL-6, IL-1β, and TNF-α, while raising the level of IL-10 in the colon (ERCs-treated group *vs*. untreated group: IL-6, P < 0.0001; IL-1β, P < 0.0001; TNF-α, P < 0.0001; IL-10, P < 0.001). These results indicated that CD73-expressing ERCs could enhance the proportion of anti-inflammatory T cell subsets and local cytokine profiles, contributing to the recovery of the intestinal barrier and thereby alleviating colitis.

### CD73^-/-^ERCs failed to inhibit DC maturation in colitis mice

**Figure 6:**
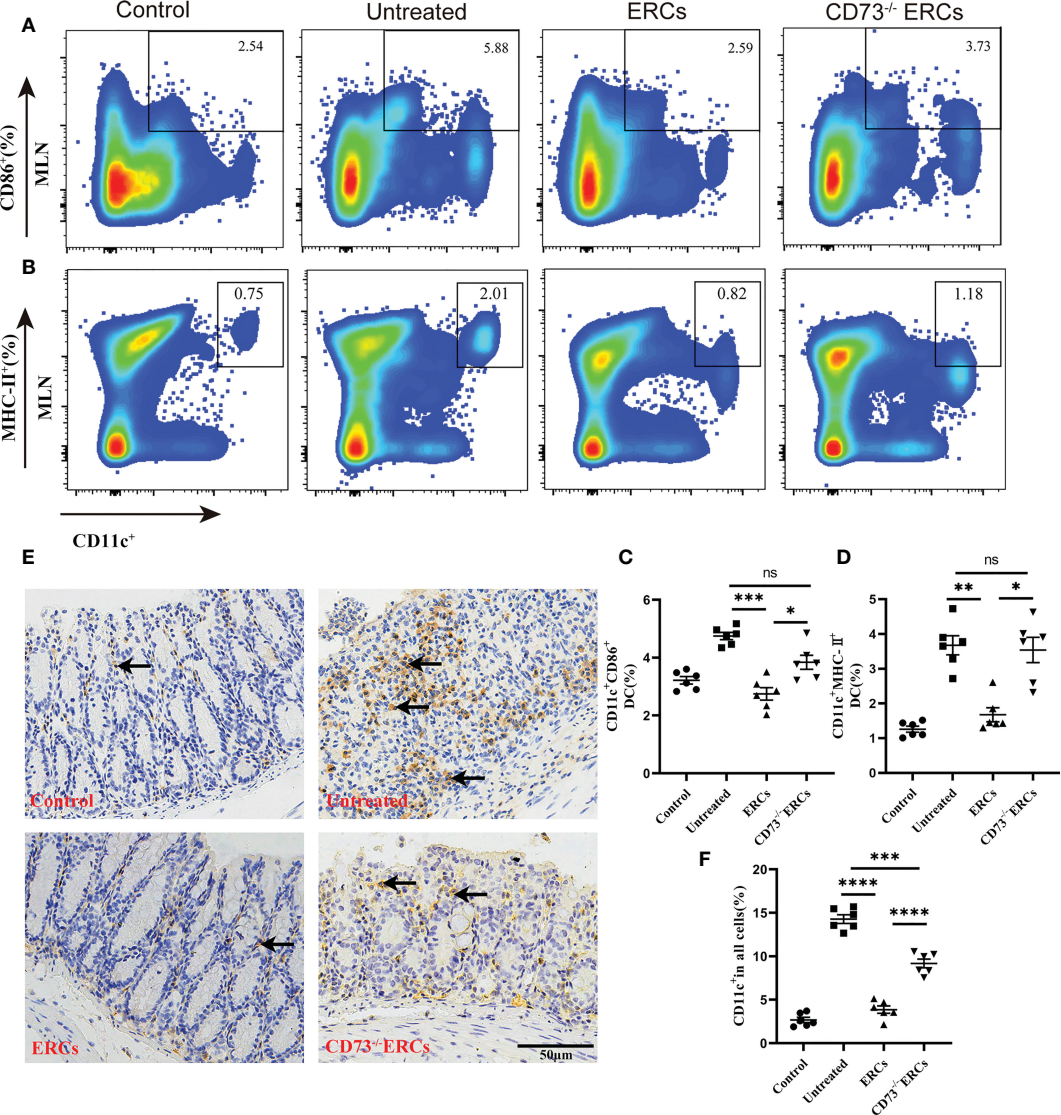
Knockout of CD73 reverses the effect of ERCs on preventing DC maturation in colitis. To determine whether each treatment affects DC phenotypes, anti-CD11c antibody and antigen presenting-related antibodies (anti-MHCII, anti-CD86) were used to measure mature DCs in MLNs. **(A, B)** Representative dot plots of CD11c^+^CD86^+^ DCs and CD11c^+^MHCII^+^ DCs in MLNs. **(C, D)** Percentage of CD11c^+^CD86^+^ DCs and CD11c^+^MHCII^+^ DCs, respectively (n=6). **(E)** Infiltration of CD11c^+^ cells was determined by immunohistochemical staining in the colon tissues of each group (Scale bar: 50 μm). **(F)** Percentage of CD11c^+^ cells by Image J analysis (n = 6). Data are presented as mean ± s.e.m (SEM). ns, no significance; *P < 0.05; **P < 0.01; ***P < 0.001; ****P <0.0001, analyzed by one-way ANOVA with LSD multiple comparison *post hoc* test.

Many studies have unambiguously proven the immunosuppressive impact of the “CD73-ADO” axis on DCs, which have been characterized as antigen-presenting cells to mediate T cell differentiation (41). Differentiated CD4^+^ T cells in MLNs were significantly improved in the CD73-expressing ERCs-treated group, which prompted us to investigate the effect of CD73 on DCs and the associated molecular mechanisms *in vivo*. To determine whether the number and function of DCs are regulated by CD73 expressed on ERCs, we conducted flow cytometry analysis using MLNs in colitis mice. **Figures 6A-D** showed that the proportion of mature DCs (CD11c^+^CD86^+^ and CD11c^+^MHC II^+^) in ERCs-treated group was remarkably lower than that of untreated group (CD11c^+^CD86^+^, P < 0.001; CD11c^+^MHC II^+^, P < 0.01). Nevertheless, knockout of CD73 on ERCs significantly increased the proportion of mature DCs compared to that of ERCs-treated group (CD11c^+^CD86^+^, P<0.05; CD11c^+^MHC II^+^, P<0.05). Furthermore, immunohistochemistry of colonic tissue stained with the marker CD11c showed that CD73 expressed on ERCs decreased intra-colon infiltration of DCs in DSS-induced colitis mice (**Figures 6E, F**, P<0.0001). These data suggested that CD73 is critical to ERCs mediated inhibition of DC maturation and protection against colitis in mice.

### CD73-expressing ERCs inhibited the antigen presentation and stimulatory function of DCs associated with the STAT-3 pathway

**Figure 7:**
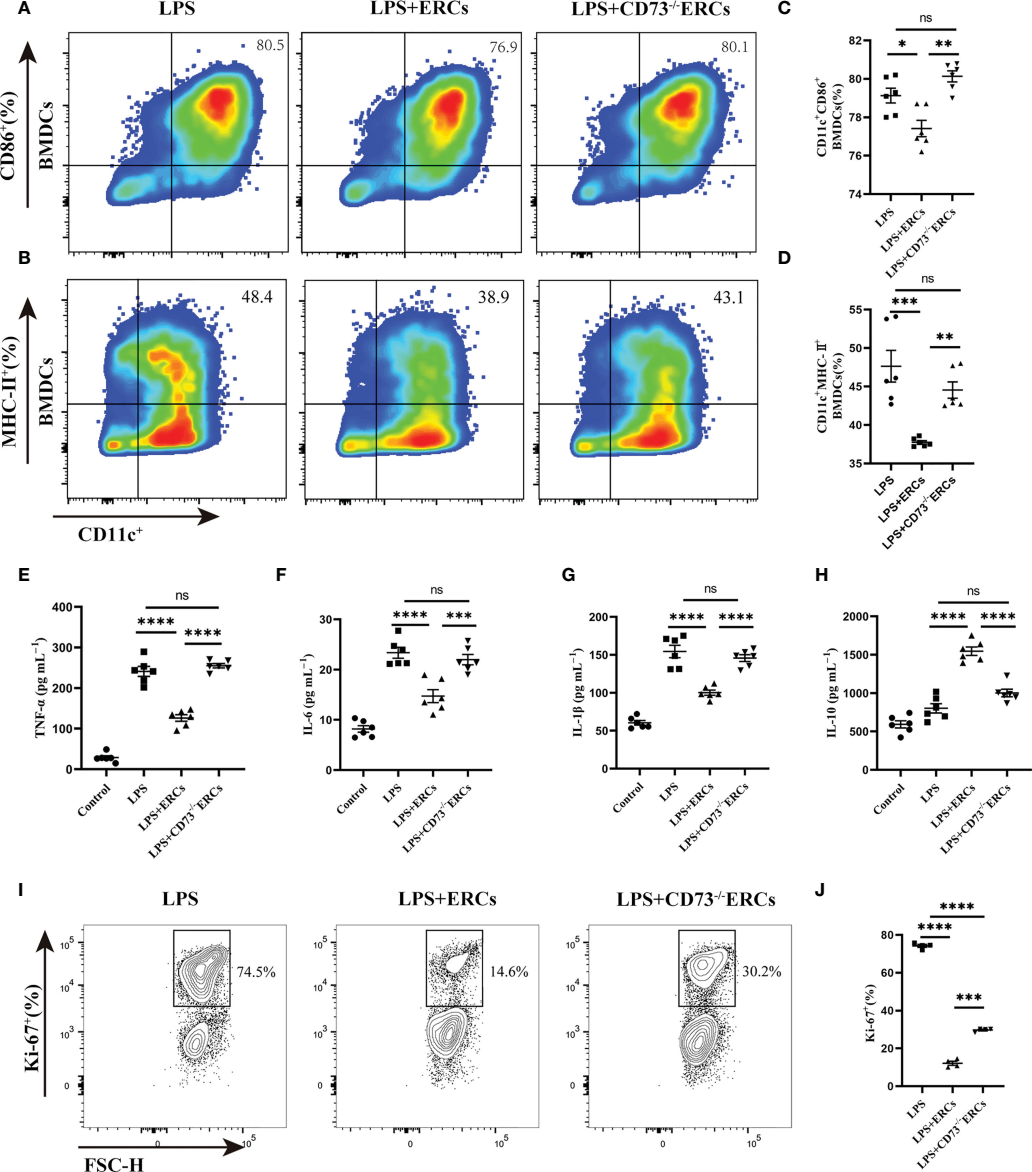
Knockout of CD73 reverses the effect of ERCs on decreasing the percentage of mature DCs and enhancing the tolerogenic function of the DCs *in vitro*. ERCs and CD73^-/-^ERCs were co-cultured with immature DCs obtained from the bone marrow of BALB/c mice with 200ng/ml LPS for 24 hours in a transwell system. The percentage of CD11c^+^CD86^+^ DCs and CD11c^+^MHCII^+^ DCs was measured by flow cytometry analysis. **(A, B)** Representative dot plots of CD11c^+^CD86^+^ DCs and CD11c^+^MHCII^+^ DCs. **(C, D)** Percentage of CD11c^+^CD86^+^ DCs and CD11c^+^MHCII^+^ DCs (n=6). **(E-H)** The IL-6, TNF-α, IL-1β, and IL-10 were examined in the supernatant of co-cultured cells (n=6). **(I)** Contour plot of Ki-67^+^ T cells *in vitro.* **(J)** Percentage of Ki-67^+^ cells (n=4). Data are presented as mean ± s.e.m (SEM). ns, no significance; * P < 0.05; **P < 0.01; ***P < 0.001; ****P <0.0001, analyzed by one-way ANOVA with LSD multiple comparison *post hoc* test.

To further understand the activity of DCs in colitis, we studied the immunomodulatory capacity of CD73-expressing ERCs on BMDCs and T cell proliferation. The immunosuppressive ability of CD73-expressing ERCs against BMDCs was activated by LPS in a co-culture system. The expression of CD86 and MHC-II on BMDCs were assessed to evaluate the impact of CD73 on ERCs. It was found that ERCs significantly dampened the activation of BMDCs, characterized by decreased expression of CD86 and MHC II (**Figures 7A-D**, CD11c^+^CD86^+^, P<0.05; CD11c^+^MHC II^+^, P<0.001). In contrast, the suppression of BMDC maturation by ERCs was diminished by the deletion of CD73 (**Figures 7A-D**, CD73^-/-^ERCs-treated group *vs*. ERCs-treated group: CD11c^+^CD86^+^, P<0.01; CD11c^+^MHC II^+^, P<0.01). In addition, CD73-expressing ERCs elevated the level of IL-10 while depressing the concentrations of TNF-α, IL-6, and IL-1β (**Figures 7E-H**, ERCs-treated group *vs*. untreated groups, P < 0.0001), suggesting its ability to inhibit the activation of pro-inflammatory cells. Next, we verified the stimulatory ability of DCs to induce T cell proliferation with a co-culture system. As shown in **Figure 7I, J**, the proliferation index of CD4^+^ T cells was significantly reduced in the LPS+ERCs group compared to that of the LPS group (P < 0.0001), but the proliferation index was markedly increased in the CD73^-/-^ERCs-treated group (LPS+ERCs group *vs*. LPS+CD73^-/-^ERCs group, P < 0.001). These data suggest that CD73 mediates ERC-induced inhibition of antigen presentation and T cell stimulation by DCs, and inhibits DC maturation.

**Figure 8:**
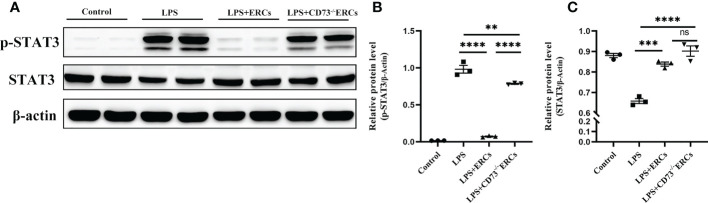
Knockout of CD73 reverses the effect of ERCs on the tolerogenic function of the DCs associated with the STAT-3 pathway *in vitro*. To explore whether STAT3 would participate in DC maturation and activation mediated by CD73 expressing ERCs *in vitro*, western blot was used to determine p-STAT3 and STAT3 with β-actin as a reference in DCs (A-C, n=3). Data are presented as mean ± s.e.m (SEM). ns, no significance; **P < 0.01; ***P < 0.001; ****P <0.0001, analyzed by one-way ANOVA with LSD multiple comparison *post hoc* test.

We speculated that the signal transducer and activator of transcription 3 (STAT3) could be involved in the ERC-mediated regulation of DCs. Therefore, we next explored whether STAT3 participated in DC maturation and activation mediated by CD73 expressing ERCs *in vitro*. Notably, as shown in **Figures 8A-C**, p-STAT3 was elevated in response to LPS stimulation, consistent with previous findings that STAT3 is essential for the maturation of DCs. The p-STAT3 and STAT3 normalized within the ERCs group (LPS+ERCs group *vs*. LPS group: p-STAT3, P<0.0001; STAT3, P<0.001). In contrast, CD73 knockout significantly impaired the contribution of ERCs (LPS+CD73^-/-^ERCs group *vs*. LPS+ERCs group: p-STAT3, P<0.0001). Together, these results indicate that STAT3 is a positive regulator for DC maturation and function, and CD73-expressing ERCs can manipulate STAT3 activity in DCs to control the colitis progress.

## Discussion

IBD is a chronic, idiopathic and relapsing enteric disorder, which is believed to result from an abnormal immune response to the intestinal flora due to epithelial barrier dysfunction in a susceptibility genetic background (42). With the broadened recognition of the pathogenesis of IBD, various novel effective treatments have emerged. Stem cell therapy with MSCs has generated significant interest among them, but limited resources and invasive isolation procedures restricted the widespread application of these cells (43). As a novel source of MSCs, human ERCs are free from the drawbacks of traditional invasive harvesting methods and possess their advantages, including abundant resources, non-invasive harvesting procedures, and feasibility of bulk production (44). Previous studies have shown that ERCs could be chemotactic to inflammatory sites and exert immunomodulatory properties to mediate the alleviation of colitis (15). Nevertheless, the exact mechanism of ERCs remains to be elucidated.

Our findings demonstrated that ERCs were a prolific candidate for IBD management that provide a CD73^+^ cellular environment locally by intraperitoneal injection in the inflammation process, which facilitates the hydrolysis of pro-inflammatory ATP into immunosuppressive ADO. Notably, male mice were chosen as animal models in this study to maintain the uniformity and stability of the experimental results. CD73-expressing ERCs effectively attenuated body weight loss, bloody stool, shortening colon length, and pathological damage. Knockout of CD73 impaired the restoration of ERCs to colonic goblet cells, ultrastructure of tight junctions and microvilli, intestinal permeability and associated tight junction protein expression. Surprisingly, CD73-expressing ERCs significantly decreased the populations of Th1 and Th17 cells but increased the proportions of Tregs in the mesenteric lymph nodes. Furthermore, CD73-expressing ERCs markedly reduced the levels of pro-inflammatory cytokines (IL-6, IL-1β, TNF-α) and increased anti-inflammatory factors (IL-10) levels in the colon. Significantly, CD73-ERCs regulate DC maturation and activation *via* the STAT3 pathway, regulate innate immune responses, and exert efficient therapeutic efficacy against colitis. These data all together shed light on insight into the mechanisms by which ERCs regulate purine metabolism *via* CD73 that might be involved in minimizing the dysbiosis associated with the pathogenesis of IBD.

Among the phenotypic markers, CD73 in ERC functions has been largely ignored. CD73 was the rate-limiting enzyme in purinergic signaling with the final step converting AMP to ADO. Studies have increasingly demonstrated by studies to be involved in cellular homeostasis, physiological adaptations, and pathological processes (23, 45). The enzymatic degradation of exogenous pro-inflammatory ATP to anti-inflammatory ADO has been regarded as pivotal in regulating the local inflammatory environment and has systemic implications (23). ADO reduced the activation of DCs, macrophages, NK, B, and CD4^+^ T cells, and the accumulation of neutrophils while facilitating the suppression of DC maturation and the generation of M2 macrophages and Treg cells, thus instituting an adenosinergic augmentation loop (46–49). It was further shown to be a powerful systemic immunosuppressor, as ADO production was used as an immune escape strategy by pathogens (50, 51) and tumor cells (52, 53), and its accumulation caused a severe combined immunodeficiency lacking adenosine deaminase (ADA) for Ado degradation (54). In this study, immunoblotting and free Pi concentrations demonstrated that lentiviral transfection completely blocked CD73 expression on ERCs. Furthermore, the deletion of CD73 significantly weakened the effect of ERCs in protecting intestinal barrier function and regulating the local immune response in the murine colitis model.

CD73 expression was also found aberrantly upregulated in numerous types of tumor microenvironments, such as colorectal, gastric, hepatocellular and ovarian cancers (55–58). Studies have shown that CD73/adenosine signaling is associated with tumorigenesis and tumor progression through inhibition of CD4^+^T cells and NK cell proliferation, while augmenting suppressive immune subsets such as Tregs, regulatory B cells, and myeloid-derived suppressor cells (59, 60). Besides immune-related mechanisms, Wu R et al. found that CD73 also promoted the proliferation of colorectal cancer cells through EGFR and β-catenin/cyclin D1 signaling pathways (61). Metastasis as a vicious characteristic of colorectal cancers, CD73 is strongly associated with tumor metastasis in both experimental models and clinical patients (62, 63). CD73 linked adenosine signaling in tumor microenvironmental switching to epithelial-to-mesenchymal transition phenotype by suppressing phosphorylation of LIMK/cofilin and facilitating activation of β-catenin during tumor metastasis (56, 64). High density of abundant angiogenesis is essential for the growth and metastasis of colorectal cancer. Accordingly, Allard D and colleagues showed that expression of CD73 on cancerous and host cells promotes angiogenesis process in part through induction of VEGF (65). CD73 reduced collagen IV adhesion by endothelial cells and promoted migration. Thus, CD73 has become a novel therapeutic target with its function in tumor cell proliferation, angiogenesis and metastasis of colorectal cancer.

Choosing an optimized route of drug administration was essential in stem cell therapy (66). Although intravenous injection (i.v.) was one of the most common approaches to MSC delivery, the “first-pass” effect in the lungs can lead to severe cell entrapment (67). Once injected intravenously, MSCs were trapped in the lung barrier due to their large size and cleared by monocytes/macrophages within hours, thus theoretically hindering the effect of MSCs on targeting inflammatory tissues (68, 69). Previous studies reported that MSCs injected intraperitoneally showed better therapeutic efficacy in IBD compared to *i.v.* injection, as intraperitoneal injections (i.p.) were free of cell entrapment and risk of pulmonary embolism (70). Consistent with the present study, we found that i.p. as a drug delivery modality for CD73-expressing ERCs may be more conducive to targeting inflammatory tissues. In this study, CD73-expressing ERCs-based therapy significantly ameliorated the signs of DSS-induced colitis, including body weight loss, shortened colon length, hematochezia, and pathological scoring in mice; the finding is in accordance with the report from our previous studies (71).

In the intestinal epithelium, CD73 is highly expressed on apical enterocytes. It can neutralize ATP released by bacteria in the enteric lumen, thus regulating the inappropriate immune response of the host microbiota (72). Synnestvedt K, et al. found that the CD73 inhibitor α, β-methylene ADP significantly increased intestinal permeability, demonstrating that CD73 was involved in the protective pathway for the maintenance of epithelial barrier function (73). The intestinal barrier is a defender against pathogen intrusion, and intestinal barrier dysfunction causes colitis (74). The mechanical barrier consists of the mucus layers and TJ of the intestinal epithelial cells (IECs). Mucus is generated and secreted by the goblet cells in IECs and facilitates the protection of IECs (75). Based on this, we proposed that ERCs may increase the concentration of CD73 in the local microenvironment to restore the intestinal barrier in colitis. Consistent with the current study, CD73-expressing ERC treatment remarkably rescued the goblet cell. ZO-1, Occludin, and Claudin-1 were pivotal members of the TJ transmembrane and cytoplasmic plaque protein family, which were essential for maintaining the integrity and function of the intestinal barrier (76). Encouragingly, intraperitoneal injection of CD73-expressing ERCs increased expression of the TJ proteins in colon tissue when compared with CD73^-/-^ERCs, and this phenomenon could also be visualized directly under TEM. Additionally, disruption of the intestinal epithelial barrier increased intestinal permeability and facilitated pathogenic invasion (77). Our results showed that ERCs significantly restored intestinal barrier function in mice with colitis, in which the CD73^+^ cellular environment is necessary.

However, dysregulated T cell responses and abnormal subpopulation differentiation of activated T cells might induce the onset of inflammation through the excessive release of cytokines that have multiple pathogenic effects on intestinal homeostasis (78). The heightened adaptive immune disorder seen in IBD appeared to be related to aberrant purinergic signaling (6). Concerning the significant therapeutic effect of CD73-expressing ERCs on colitis, we further evaluated its immune modulation role in CD4^+^ cell differentiation. CD73-expressing ERCs significantly decreased the populations of Th1 and Th17 cells but increased the proportions of Tregs in the MLN. The mechanism may be related to the provision that ERCs provide a CD73^+^ cellular environment locally in inflammation, which facilitates the metabolism of ADO (79). Extracellular pro-inflammatory ATP can be released in inflammatory and tissue damage microenvironment, triggering a cascade of the secondary strike (80). Feriotti C et al. reported that the ATP receptors-P2X7R activated pyrin domain containing-3 protein (NLRP3), which mediated Th1/Th17 immunity (81). Pharmacological antagonism of P2X7R promoted the spontaneous conversion of CD4^+^ T cells to Tregs after TCR stimulation (82). CD73 orchestrates CD4^+^ T cell differentiation by purinergic metabolic reprogramming (83). Borg N et al. found that CD73-encoding gene (NT5E) deletion skewed T cells toward Th1 and Th17 subsets in CD73^-/-^mice, increasing their respective pro-inflammatory cytokines IFN-γ and IL-17 (84). These pro-inflammatory cytokines induced a positive feedback loop leading to neutrophil recruitment to the inflammatory sites, enterocyte apoptosis, and maintained a pro-inflammatory milieu, which resulted in excessive tissue destruction (85). Importantly, as the enzymatic product of CD73, ADO has been proven to increase the proportion of Tregs and further enhance its immunomodulatory activity (86). The absence of the ADO receptor A2aR in Tregs reduced their immunosuppressive efficacy *in vivo* (87). The above theory explains the ability of ERCs to decrease Th1/Th17 because ERCs increase the local CD73 content, which facilitates the hydrolysis of pro-inflammatory ATP released locally by inflammation. In contrast, ADO, the product of hydrolysis by CD73, promotes the differentiation of Tregs.

Due to large amounts of dietary and microbial antigens, DCs played a pivotal role in maintaining intestinal homeostasis and suppressing inflammation caused by multilevel disruptions of intestinal homeostasis (88). CD73^+^ cellular environment is integral to the “steady state” immunosuppressive mechanism in DCs (41). Genetic ablation of CD73 in mice leads to an enhanced inflammatory response in autoimmune diseases driven by increased maturation and migration of local DCs to peripheral lymph nodes (89). Importantly, as an enzymatic product of CD73, ADO has been proven to increase the proportion of immature DCs in the inflammatory site and promote the immune tolerance of mature DCs. Consistently, our study found that the expression of CD73 is critical for ERCs to reduce the population of mature DCs and enhance the tolerogenic function of DCs. Many reports showed that DCs could express ADO receptor subtypes in varying degrees (90). Immature DCs express A1aR and A3aR, activated after recruiting immature DCs to inflammatory sites (91). While A2aR was highly expressed after DC maturation, local ADO triggered rather inhibitory effects through A2aR, such as reducing the secretion of inflammatory factors and polarizing T cell immune tolerance (92). We infer that ERCs may increase the concentration of ADO in the inflammatory microenvironment through AMP and ATP dephosphorylation. Under high ADO conditions in the inflammatory microenvironment, DCs shift to a novel tolerogenic function, which hardly activates T cells and inhibits the production of pro-inflammatory cytokines. Further *in vitro* studies also demonstrated the maturation-inhibiting effect of ADO on DCs. In addition, we also observed that CD73 expressing ERCs promoted the secretion of the anti-inflammatory factor IL-10 by DCs, while IL-6, TNF-α, and IL-1β secretion were reduced in an *in vitro* co-culture system.

The signal transducer and activator of transcription 3 (STAT3), among all members of the STAT family, has been well established as an effective therapeutic target for IBD (93). Furthermore, inhibition of STAT3 activation not only maintains and strengthens the epithelium’s barrier function but also inhibits the maturation and differentiation of immune cells, such as DCs, macrophages, and T cells (94, 95). At the molecular level, we demonstrated that CD73-expressing ERCs inhibited STAT3 phosphorylation in DCs, consistent with their secretion of IL-10 and inhibition of T cell activation. This was in agreement with the finding by Barton et al., who proved that the stimulatory ability of DCs was correlated with the level of STAT3 activation (96). Similarly, STAT3 signaling was constitutively activated in tumor-infiltrating immune cells, including DCs, and ablation of STAT3 triggered immune cells to suppress tumor proliferation and metastasis (97). However, other signaling pathways, such as the PI3K/Akt, could also be involved in DC inhibition by CD73/ADO. AdoRs have multiple molecular targets, and the overall immunosuppressive effect is obvious. It has been reported that ADO can significantly induce DC immunosuppression and tolerance phenotype. The intracellular suppressive pathways triggered by A2aR types suppress downstream PI3K/AKT signaling pathways leading to reduced mTORC activity either directly or *via* PRAS40 (41). Furtherly, Chen D, et al. demonstrated that ERCs can attenuate the ALF-induced liver injury *via* inhibition of *via* regulation of PI3K/Akt/mTOR/IKK pathway. Then, this inhibited the p-NF-κBp65 translocation to the nucleus, which contributes to a diminished release of inflammatory factors. Moreover, A2AR agonists could act synergistically with ERCs to enhance these effects (98). Thus, other potential mechanisms of its CD73-expressing ERC are to be further investigated *in vitro* and in animal experiments.

Our findings indicated that the expression of CD73 on ERCs could improve intestinal barrier function and modulate the immune response in the murine colitis model, and the process is associated with STAT3-mediated DCs. CD73 appears to play a pivotal role in the immunomodulatory effect of ERCs on DSS-induced colitis. However, ADO is known to bio-bind to at least four forms of receptors. The specific receptor(s) that mediate the effects of ADO on DCs remains to be identified. We believe that coming studies that will utilize mice conditional deletion of various receptors that interact with ADO in monocytes and/or DCs will provide better opportunities to fully illuminate the signal pathway(s) in DCs. Furthermore, the present study is limited to a mouse model of DSS-induced acute colitis and needs to be verified in other preclinical models of IBD and patients with IBD. Taken together, translating these findings to the therapeutic effects of ERCs will be essential for better understanding the pathogenesis of IBD and will guide translational and clinical efforts for the treatment of IBD. During this process, the CD73-expressing ERCs-based therapy plays a central role in determining clinical outcomes.

## Conclusion

The present study has demonstrated that ERCs, as a generalizable therapeutic approach for IBD management, provide a CD73^+^ cellular environment with depleted pro-inflammatory ATP and promote the generation of immunosuppressive ADO. CD73-expressing ERCs significantly restored the epithelium barriers, modulated immune response decreased the Th1 and Th17 subsets, and markedly improved cytokine profiles in colon tissues. Importantly, CD73-expressing ERCs are associated with the generation of immature DCs, attenuation of immune responses, and the potential therapeutic effect against colitis. Taken together, this study offers a novel insight into the immunomodulatory mechanisms of ERCs and will guide translational and clinical work in the treatment of IBD.

## Data availability statement

The original contributions presented in the study are included in the article/supplementary material. Further inquiries can be directed to the corresponding author.

## Ethics statement

The studies involving human participants were reviewed and approved by the Medical Ethics Committee of Tianjin Medical University General Hospital (IRB2021-KY-347, Tianjin, China). The patients/participants provided their written informed consent to participate in this study. The animal study was reviewed and approved by The Animal Care and Use Committee of Tianjin Medical University (IRB2021-DWFL-405, Tianjin, China).

## Author contributions

BS: Conception and design, collecting and assembling data, analyzing and interpreting data, writing manuscripts. S-HR and Z-BW: Collecting and assembling data, data analysis, and interpretation, writing manuscripts. H-DW, J-YZ, HQ, Y-LZ, C-LS, Y-NX, and XL: Collecting and assembling data, data analysis, and interpretation. HW: Conception and design, administrative support, financial support, manuscript writing and final approval of the manuscript. All authors contributed to the article and approved the submitted version.
